# Predictive value of maternal serum placental growth factor levels for discordant fetal growth in twins: a retrospective cohort study

**DOI:** 10.1186/s12884-023-06212-1

**Published:** 2024-01-02

**Authors:** Shuai Li, Kaiqi Wu, Shaomin Zhou, Binbin Yin, Xiaoxia Bai, Bo Zhu

**Affiliations:** 1 Department of Clinical Laboratory, Women’s Hospital, School of Medicine, Zhejiang University, 1 Xueshi Road, Hangzhou, 310006 China; 2 Department of Clinical Laboratory, Jilin Hospital of Women’s Hospital, School of Medicine, Zhejiang University, 555 Xiwuma Road, Changchun, 130042 China; 3 Department of Obstetrics and Gynecology, Women’s Hospital, School of Medicine, Zhejiang University, 1 Xueshi Road, Hangzhou, 310006 China; 4 Traditional Chinese Medicine for Reproductive Health Key Laboratory of Zhejiang Province, 1 Xueshi Road, Hangzhou, 310006 China; 5 Zhejiang Provincial Clinical Research Center for Obstetrics and Gynecology, 1 Xueshi Road, Hangzhou, 310006 China; 6 Key Laboratory of Women’s Reproductive Health, 1 Xueshi Road, Hangzhou, 310006 China

**Keywords:** Discordant fetal growth, Monochorionic twin pregnancy, Dichorionic twin pregnancy, Placental growth factor, Receiver operating characteristic

## Abstract

**Background:**

Accurate prenatal recognition of discordant fetal growth in twins is critical for deciding suitable management strategies. We explored the predictive value of the level of maternal second-trimester placental growth factor (PLGF) as a novel indicator of discordant fetal growth.

**Methods:**

A total of 860 women pregnant with twins were enrolled, including 168 women with monochorionic twins (31 cases of discordant fetal growth and 137 without) and 692 with dichorionic twins (79 cases of discordant fetal growth and 613 without). Maternal second-trimester PLGF concentrations were measured via immunofluorescence.

**Results:**

Maternal second-trimester PLGF levels were significantly lower in women pregnant with twins who subsequently developed discordant fetal growth than in those who did not (monochorionic twin pregnancy: *P* < 0.001; dichorionic twin pregnancy: *P* < 0.001). A 3–4 fold difference in median PLGF concentrations was detected between the two groups with both monochorionic and dichorionic twin pregnancies. Maternal second-trimester PLGF levels were significantly correlated with birth weight differences (monochorionic twin pregnancy: *r* =  − 0.331, *P* < 0.001; dichorionic twin pregnancy: *r* =  − 0.234, *P* < 0.001). A receiver operating characteristic curve was used to evaluate the predictive efficiency. In monochorionic twin pregnancies, the area under the curve (AUC) was 0.751 (95% confidence interval [CI]: 0.649–0.852), and the cutoff value was 187.5 pg/mL with a sensitivity of 77.4% and specificity of 71.0%. In dichorionic twin pregnancies, the AUC was 0.716 (95% CI; 0.655–0.777), and the cutoff value was 252.5 pg/mL with a sensitivity of 65.1% and specificity of 69.6%. Based on the above cutoff values, univariate and multivariate logistic regression analyses were performed to calculate the odds ratios (OR) for the PLGF levels. After adjustment for potential confounding factors, low PLGF concentrations still significantly increased the risk of discordant fetal growth (monochorionic twin pregnancy: adjusted OR: 7.039, 95% CI: 2.798–17.710, *P* < 0.001; dichorionic twin pregnancy: adjusted OR: 4.279, 95% CI: 2.572–7.120, *P* < 0.001).

**Conclusions:**

A low maternal second-trimester PLGF level is considered a remarkable risk factor and potential predictor of discordant fetal growth. This finding provides a complementary screening strategy for the prediction of discordant fetal growth and offers a unique perspective for the subsequent research in this field.

## Background

Recently, the incidence of twin pregnancies has increased significantly owing to advanced maternal age and the use of assisted reproductive technologies. The incidence of twin pregnancies was 32.1 per 1000 births in the United States and 15.3 per 1000 births in the UK, of which monochorionic pregnancies account for about 30% and dichorionic pregnancies for about 70% [1–3]. Twin pregnancy is associated with severe maternal complications and adverse perinatal outcomes. Discordant fetal growth is a major determinant of perinatal outcomes in twin pregnancies. According to the American College of Obstetricians and Gynecologists practice bulletin on multiple gestations, discordant fetal growth is defined as a 20% difference in fetal weight between the larger and smaller fetuses; this discordance is associated with intrauterine growth restriction, stillbirth, preterm birth, fetal abnormality, admission to the neonatal intensive care unit, and respiratory distress [4–7].

Considering the adverse perinatal outcomes of discordant growth, accurate prenatal prediction and recognition of discordant growth are crucial. Currently, comparing fetal biometry parameters via ultrasound to evaluate the degree of inter-twin fetal growth discordance has become a routine obstetric practice [8–10]. The predictive value of fetal growth parameters include crown-lump length (CRL), biparietal diameter (BPD), head circumference (HC), abdominal circumference (AC), and femur length (FL). Estimated fetal weight (EFW) is calculated using empirical formulas based on the above parameters [11–13]. However, some disadvantages of conventional predictive indicators cannot be overlooked. First, the parameters obtained via ultrasound can be affected by many factors, including the sonographer’s experience, fetal position, amniotic fluid volume abnormalities, fetal subcutaneous fat thickness, and differences in formulas. Furthermore, establishing an accurate prediction of birth weight discordance using these parameters is challenging owing to the presence of positive and negative biases in EFW, e.g., a maximum error of 20% may be estimated if one fetus’ weight is overestimated by 10% and the other’s weight is underestimated by 10%. Therefore, novel indicators, especially more objective serological biomarkers, should be explored.

Placental growth factor (PLGF), a proangiogenic protein, is a member of the vascular endothelial growth factor family and is produced by villous syncytiotrophoblasts in the placenta [14]. Altered PLGF levels may be observed under abnormal placentation, leading to insufficient remodeling of the maternal spiral arteries and placental ischemia. Previous studies have confirmed that PLGF levels in the maternal blood circulation are altered in pregnancies complicated by preeclampsia, fetal growth restriction, preterm delivery, or stillbirth [15–18]. Considering that placental abnormalities are one of the main pathological mechanisms of discordant growth in twins, changes in PLGF levels may be observed in women pregnant with twins who subsequently develop discordant fetal growth and can even be used to predict the presence of discordant growth. However, a few studies have focused on this issue. In the study by Kadioglu SG et al., which included 79 pairs of twins, a significant correlation was observed between birthweight discordance and PLGF level (*r* = 0.430, *P* = 0.001). Nevertheless, Mackie FL et al. (*n* = 177) found that there was no statistically significant association between antenatal growth restriction (growth discordance > 20%) and first-trimester PLGF level in monochorionic twins (adjusted OR 0.88, 95% CI: 0.44–1.76, *P* = 0.720) [19, 20].

Thus, this study aims to determine whether maternal second-trimester PLGF levels are associated with discordant growth in twins and to evaluate the predictive value of PLGF levels for discordant fetal growth in monochorionic and dichorionic pregnancies.

## Methods

### Participants

**Fig. 1:**
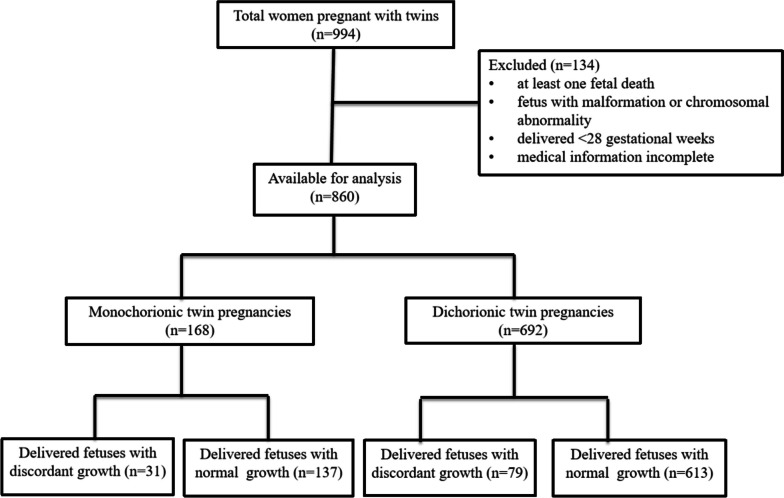
Study workflow of the present study

This study was conducted at the Women’s Hospital, School of Medicine, Zhejiang University (China) between June 2021 and October 2022. Women (aged 20–45 years) pregnant with twins at 24–28 weeks of gestation, as shown on first-trimester ultrasonography, were enrolled. All patients underwent prenatal examinations and delivered at our department. The exclusion criteria included: (1) twins with unclear chorionicity, (2) at least one fetal death, (3) at least one fetus with malformation, (4) at least one fetus with a chromosomal abnormality, (5) delivery before 28 gestational weeks, and (6) incomplete medical information. The final study included 860 participants (168 monochorionic and 692 dichorionic twin pregnancies) (Fig. 1). The study protocol was approved by the Ethics Committee of the Women’s Hospital, School of Medicine, Zhejiang University (China) (IRB-20230222-R).

The data collected for each participant was maternal age; pre-pregnancy body mass index (BMI); pre-pregnancy systolic pressure; pre-gestation diastolic pressure; gravidity; parity; gestational weight gain; gestational age at delivery; mode of conception; mode of delivery; maternal obstetric complications, including gestational diabetes mellitus, intrahepatic cholestasis of pregnancy, preeclampsia, hypothyroidism along with pregnancy, anemia along with pregnancy, and premature rupture of membranes; birth weights of the larger and smaller fetuses; Apgar scores of the fetuses; neonatal anemia; and neonatal respiratory distress syndrome (NRDS).

For all participants in the second trimester (24–28 weeks), blood samples were collected in a vacuum tube (BD Vacutainer Systems) after 6–12 h of fasting, which was centrifuged at 2,000 × g for 10 min, and the PLGF concentrations were then measured via immunofluorescence on an iRaTe 1600 Immunofluorescence Analyzer (Ningbo Aocheng) within 72 h. All tests were performed using the original reagents and according to the manufacturer’s instructions.

### Judgement criteria

Birth weight discordance was evaluated using the following formula: ([larger birth weight – smaller birth weight]/larger birth weight) × 100%. Twin discordance was defined as a birth weight difference of ≥ 20% [4]. Based on these criteria, the participants were divided into two groups, the discordant growth (≥ 20% difference in birth weight) and normal growth (< 20% difference in birth weight) groups.

### Statistical analysis

Statistical analyses were performed using the SPSS 20.0 software. Statistical significance was set at a *P*-value of < 0.05. The Shapiro-Wilk test was used to verify normality. Descriptive statistics were presented as mean ± standard deviation for normally distributed variables and median (25–75th percentile) for non-normally distributed variables. Parametric (t-test) and non-parametric (Mann-Whitney *U* test) analyses were performed for normally and non-normally distributed variables, respectively. Categorical data were compared using the chi-square or Fisher’s exact tests, where appropriate. A spearman’s rank correlation analysis was used to analyze the association between the PLGF levels and differences in birth weights in twin pairs. A receiver operating characteristic (ROC) curve analysis was used to evaluate the predictive efficiency of PLGF levels for discordant growth, and the maximum Youden index value served as the cutoff value. Finally, univariate and multivariate logistic regression analyses were performed to calculate the odds ratios (OR) and 95% confidence intervals (CIs), and potential confounding factors were included.

## Results

###  Demographic characteristics of the participants

Twin pregnancies were divided into discordant growth and normal growth groups for monochorionic and dichorionic twins, respectively. The maternal and neonatal demographic data are presented in Table 1. Compared with the normal growth group, the discordant growth group comprised a higher proportion of mothers aged > 30 years (monochorionic twin pregnancy: *P* = 0.044; dichorionic twin pregnancy: *P* = 0.027). Newborns in the discordant growth group had a lower birth weight of the smaller fetus and 5-min Apgar score and a higher incidence of NRDS. In monochorionic twins, significant differences were observed in gestational weight gain, gestational age at delivery, birth weight of the larger fetus, and 1-min Apgar score (*P* = 0.037, *P* < 0.001, *P* < 0.001, and *P* < 0.001, respectively). However, no significant differences were observed for these factors in dichorionic twins (*P* = 0.923, *P* = 0.689, *P* = 0.628, and *P* = 0.287, respectively). In addition, no significant differences were observed between pre-pregnancy BMI scores, pre-gestation systolic and diastolic pressures, gravidity, and parity in both monochorionic and dichorionic twins.

**Table 1:** Maternal and neonatal characteristics of the participants

	Monochorionic twin pregnancy			Dichorionic twin pregnancy		*P*
	Discordant growth group (*n* = 31)	Normal group (*n* = 137)	*P*	Discordant growth group (*n* = 79)	Normal group (*n* = 613)	
Maternal age [n (%)]						
≤ 30 years	11 (35.48)	76 (55.47)	0.044	22 (27.85)	250 (40.78)	0.027
> 30 years	20 (64.52)	61 (44.53)		57 (72.15)	363 (59.22)	
Gravidity [n (%)]						
≤ 1	17 (54.84)	60 (43.80)	0.265	48 (60.76)	336 (54.81)	0.317
≥ 2	14 (45.16)	77 (56.20)		31 (39.24)	277 (45.19)	
Parity [n(%)]						
0	24 (77.42)	91 (66.42)	0.234	67 (84.81)	511 (83.36)	0.744
≥ 1	7 (22.58)	46 (33.58)		12 (15.19)	102 (16.64)	
Gestational weight gain (kg)	13.0 (11.0–16.0)	16.0 (12.5–20.0)	0.037	15.0 (12.0–18.0)	15.0 (12.0–18.0)	0.923
Pre-pregnancy BMI (kg/m^2^)	21.00 (20.00–23.73)	20.56 (18.95–22.00)	0.083	21.23 (19.53–22.86)	20.83 (19.23–23.16)	0.811
Gestational age at delivery (weeks)	33 (31–35)	35 (34–36)	< 0.001	36 (34–37)	36 (35–37)	0.689
ART [n(%)]	5 (16.13)	15 (10.95)	0.538	58 (73.42)	433 (70.64)	0.608
Pre-gestation systolic pressure (mmHg)	115.0 (107.0–120.0)	111.0 (106.5–120.0)	0.799	113.0 (110.0–120.0)	111.0 (108.0–120.0)	0.528
Pre-gestation diastolic pressure (mmHg)	70.0 (65.0–76.0)	70.0 (65.0–77.0)	0.821	70.0 (63.0–73.0)	70.0 (65.0–75.0)	0.362
Delivery						
Vaginal delivery [n(%)]	2 (6.45)	15 (10.95)	0.742	0 (0.00)	49 (7.99)	0.009
Cesarean section [n(%)]	29 (93.55)	122 (89.05)		79 (100.00)	564 (92.01)	
Obstetric complications						
GDM [n(%)]	3 (9.68)	27 (19.71)	0.188	20 (25.32)	129 (21.04)	0.385
ICP [n(%)]	3 (9.68)	9 (6.57)	0.465	10 (12.66)	61 (9.95)	0.455
Preeclampsia [n(%)]	8 (25.81)	23 (16.79)	0.242	19 (24.05)	111 (18.11)	0.203
Hypothyroidism [n(%)]	1 (3.23)	13 (9.49)	0.471	10 (12.66)	62 (10.11)	0.486
Premature rupture of membranes [n(%)]	1 (3.23)	10 (7.30)	0.691	9 (11.39)	80 (13.05)	0.679
Anemia along with pregnancy [n(%)]	8 (25.81)	41 (29.93)	0.649	22 (27.85)	148 (24.14)	0.472
Newborn characteristics						
Birth weight of the larger fetus (g)	1980.97 ± 475.02	2295.36 ± 405.29	< 0.001	2553.29 ± 508.76	2524.59 ± 359.61	0.628
Birth weight of the smaller fetus (g)	1422.90 ± 434.94	2110.29 ± 402.21	< 0.001	1878.61 ± 445.93	2309.76 ± 337.48	< 0.001
1-min Apgar (average score of two fetuses)	8.71 ± 1.24	9.41 ± 0.82	< 0.001	9.39 ± 0.76	9.49 ± 0.75	0.287
5-min Apgar(average score of two fetuses)	9.67 ± 0.69	9.90 ± 0.31	0.006	9.79 ± 0.64	9.91 ± 0.33	0.013
Neonatal anemia [n(%)]	6 (19.35)	21 (15.33)	0.592	8 (10.13)	59 (9.62)	0.887
NRDS [n(%)]	18 (58.06)	18 (13.14)	< 0.001	14 (17.72)	40 (6.53)	< 0.001

Data are presented as mean ± standard deviation or median (25–75th percentile) or No (%)

*BMI* Body mass index, *ART* Assisted reproductive technologies, *GDM* Gestational diabetes mellitus, *ICP* Intrahepatic cholestasis of pregnancy, *NRDS* Neonatal respiratory distress syndrome

### Comparison of maternal second-trimester PLGF levels between women pregnant with twins who subsequently developed discordant fetal growth and those who did not

**Fig. 2:**
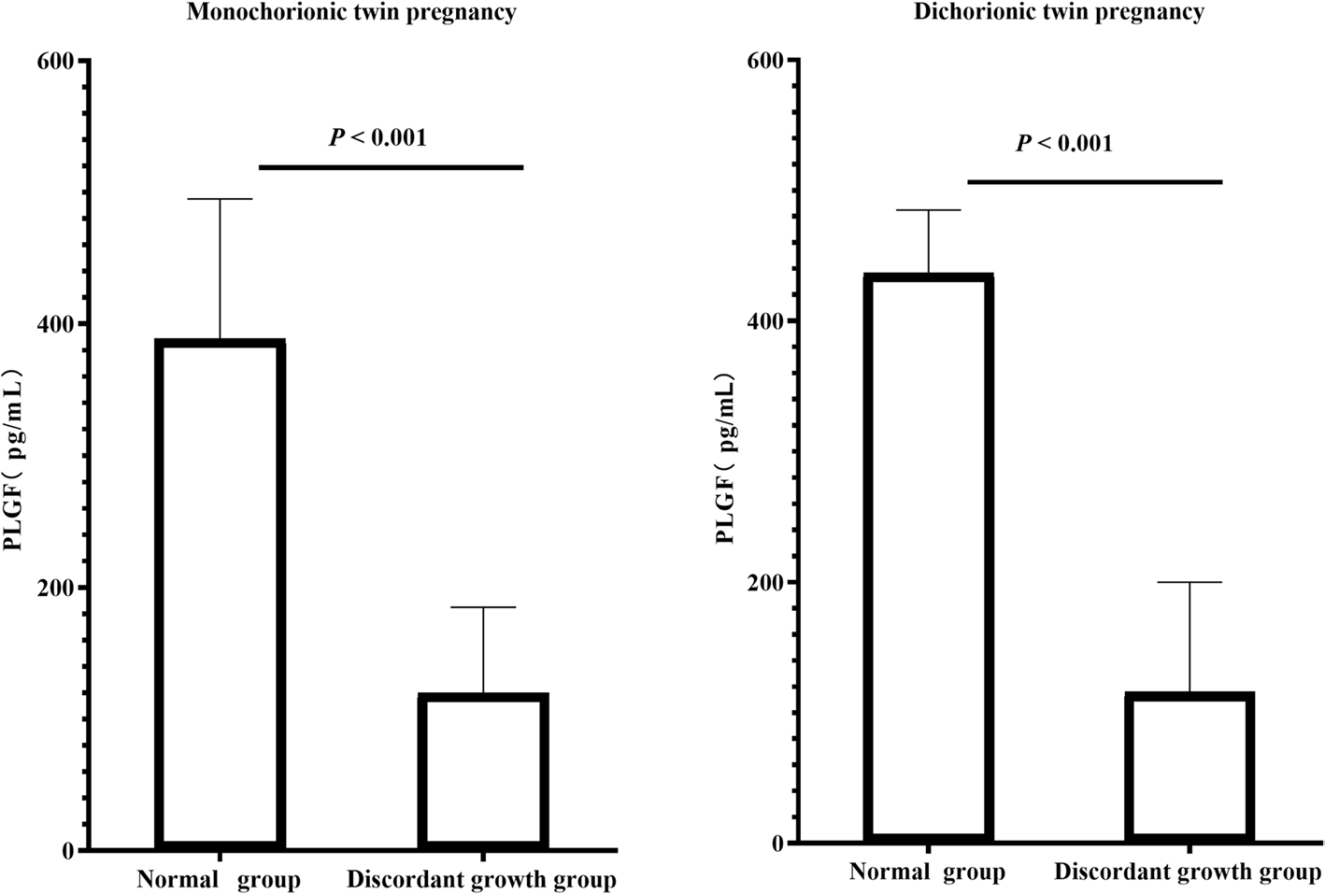
Comparison of maternal second-trimester PLGF levels between women who subsequently developed discordant fetal growth and those who did not

In monochorionic twin pregnancies, maternal second-trimester PLGF levels were significantly lower in women pregnant with twins who subsequently developed discordant fetal growth than in those who did not (*P* < 0.001). The median PLGF levels in the second trimester were 120.0 (58.0–254.0) and 389.0 (202.5–675.5) pg/mL, respectively. Similarly, in dichorionic twin pregnancies, this remarkable difference was observed (*P* < 0.001), and the median PLGF levels in the second trimester were 116.0 (53.0–349.0) and 437.0 (162.5–937.0) pg/mL, respectively. Overall, the median maternal serum PLGF concentration was 3–4 times higher in the normal fetal growth group than in the discordant fetal growth group (Fig. 2).

### Evaluation of the correlations between maternal second-trimester PLGF levels and birth weight differences

**Fig. 3:**
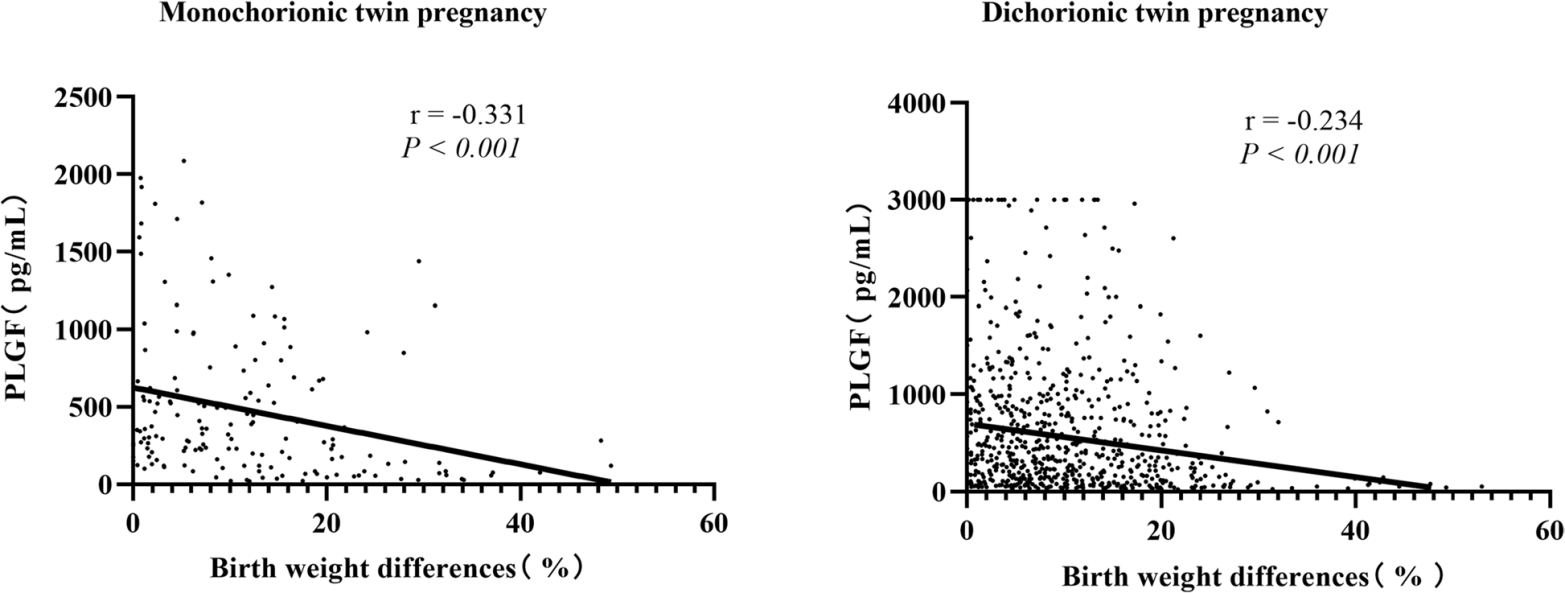
Correlations between maternal second-trimester PLGF levels and birth weight differences

First, the differences in birth weight between twin pairs were calculated according to the following formula: ([larger birth weight – smaller birth weight]/larger birth weight) × 100%, as previously described. We then investigated the correlations between maternal second-trimester PLGF levels and the differences. As shown in Fig. 3, PLGF levels were significantly correlated with birth weight differences (monochorionic twin pregnancy: *r*: − 0.331, *P* < 0.001; dichorionic twin pregnancy: r: − 0.234, *P* < 0.001). Based on this comparison, the correlation was stronger for monochorionic twin pregnancies than for dichorionic twin pregnancies. The negative correlation coefficient indicated that the lower the PLGF level, the higher the probability of developing discordant fetal growth.

### Predictive value of maternal second-trimester PLGF levels for discordant fetal growth in twins

**Fig. 4:**
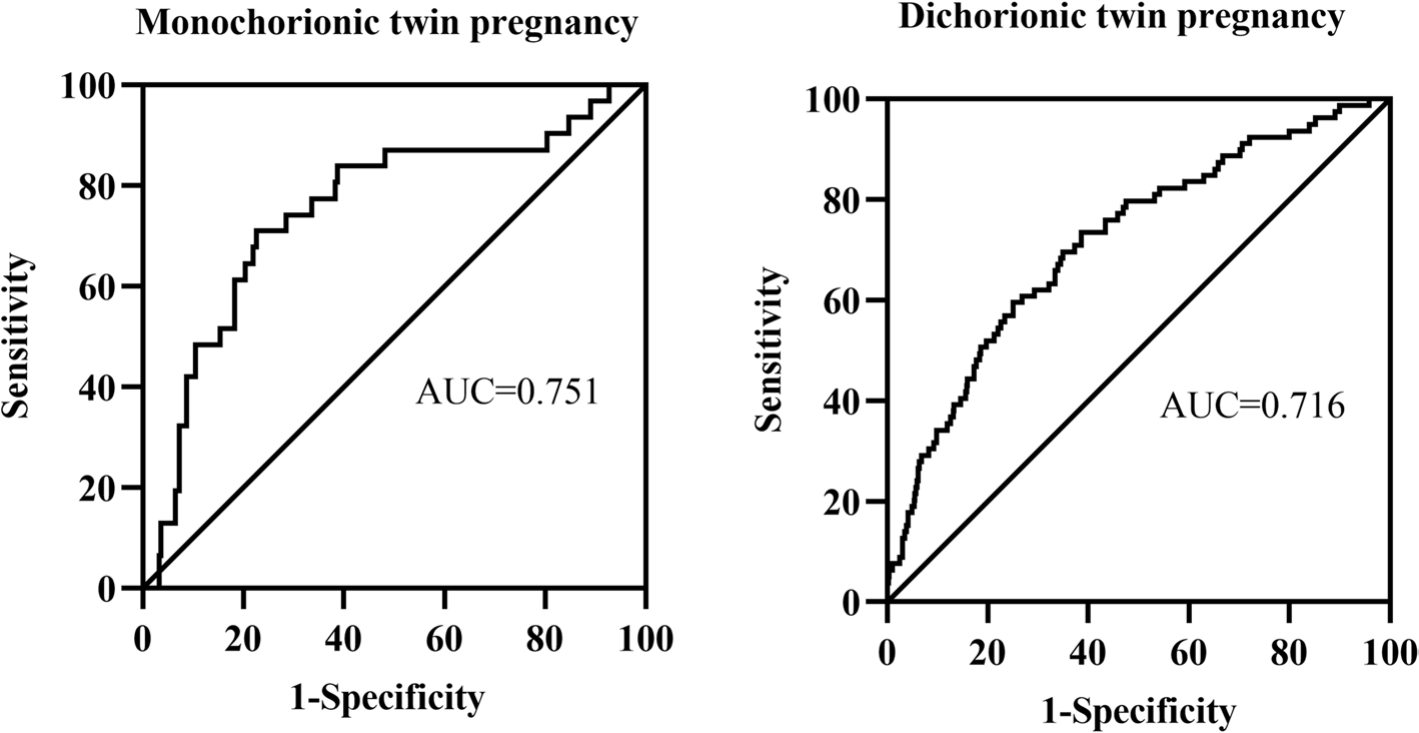
Receiver operating characteristic curves of PLGF for predicting discordant fetal growth

Given the remarkable changes in maternal serum PLGF levels in patients with discordant fetal growth and the significant correlations between PLGF levels and birth weight differences, we further evaluated PLGF as a potential biomarker for the prediction of discordant fetal growth using an ROC curve analysis. The optimal cutoff values were defined as the sum of the maximum sensitivity and specificity. In monochorionic twin pregnancies, the area under the curve (AUC) was 0.751 (95% CI: 0.649–0.852), and the cutoff value was 187.5 pg/mL with a sensitivity of 77.4% and specificity of 71.0%. In dichorionic twin pregnancies, the AUC was 0.716 (95% CI: 0.655–0.777), and the cutoff value was 252.5 pg/mL with a sensitivity of 65.1% and specificity of 69.6% (Fig. 4). Additionally, no discordant fetal growth was observed in 92.17% of pregnancies with a PLGF level of > 187.5 pg/mL (monochorionic twin pregnancy) and in 94.33% of pregnancies with a PLGF level of > 252.5 pg/mL (dichorionic twin pregnancy).

### Adjusted risk value of maternal second-trimester PLGF levels for predicting discordant fetal growth in twins

Based on the data shown in Table 1, among all the maternal characteristics, a maternal age of > 30 years, gestational weight gain, and gestational age at delivery were statistically different between the discordant fetal growth and normal growth groups in monochorionic twin pregnancies. However, only a maternal age of > 30 years differed significantly between the two groups in dichorionic twin pregnancies. Univariate and multivariate logistic regression analyses were performed to evaluate the aforementioned risk factors associated with discordant fetal growth, and the OR of PLGF levels was adjusted based on these potential confounding factors. As shown in Table 2, in the crude regression models, a maternal age of > 30 years was positively associated with discordant fetal growth in both monochorionic and dichorionic twin pregnancies (monochorionic twin pregnancy: OR: 2.265, 95% CI: 1.009–5.088, *P* = 0.048; dichorionic twin pregnancy: OR: 1.784, 95% CI: 1.063–2.994, *P* = 0.028). However, this association was not significant after adjusting for potential confounders in monochorionic twin pregnancies (adjusted OR: 1.923, 95% CI: 0.763–4.848, *P* = 0.166). Additionally, in monochorionic twin pregnancies, gestational age at delivery reduced the risk of discordant fetal growth after correcting for potential confounders (adjusted OR: 0.740, 95% CI: 0.599–0.915,* P* = 0.005); however, gestational weight gain showed no significant association with discordant fetal growth, whether adjusted or not (OR: 0.927, 95% CI: 0.856–1.005, *P* = 0.065; adjusted OR: 0.964, 95% CI: 0.882–1.055, *P* = 0.429, respectively).

**Table 2:** Univariate and multivariate logistic regression analyses of discordant fetus growth risk factors

	Monochorionic twin pregnancy				Dichorionic twin pregnancy			
	Univariate Analysis		Multivariate Analysis		Univariate Analysis		Multivariate Analysis	
	OR (95% CI)	*P*	Adjusted OR (95% CI)	*P*	OR (95% CI)	*P*	Adjusted OR (95% CI)	*P*
Maternal age [n (%)]								
≤ 30 years	Reference		Reference		Reference		Reference	
> 30 years	2.265 (1.009–5.088)	0.048	1.923 (0.763–4.848)	0.166	1.784 (1.063–2.994)	0.028	1.791 (1.055–3.042)	0.031
Gestational weight gain (kg)	0.927 (0.856–1.005)	0.065	0.964 (0.882–1.055)	0.429	NA	NA	NA	NA
Gestational age at delivery (weeks)	0.680 (0.564–0.819)	< 0.001	0.740 (0.599–0.915)	0.005	NA	NA	NA	NA
PLGF (pg/mL)								
< 187.5 (monochorionic twin)	8.358 (3.492–20.005)	< 0.001	7.039 (2.798–17.710)	< 0.001	NA	NA	NA	NA
< 252.5 (dichorionic twin)	NA	NA	NA	NA	4.273 (2.572–7.097)	< 0.001	4.279 (2.572–7.120)	< 0.001

*OR* Odds ratio, *CI* Confidence interval, *PLGF* Placental growth factor, *NA* Not applicable

In the crude regression models, twin-pregnant women with a second-trimester PLGF concentration of < 187.5 pg/mL (monochorionic twin pregnancy) or of < 252.5 pg/mL (dichorionic twin pregnancy) had an increased risk of developing discordant fetal growth (monochorionic twin pregnancy: OR: 8.358, 95% CI: 3.492–20.005, *P* < 0.001; dichorionic twin pregnancy: OR: 4.273, 95% CI: 2.572–7.097, *P* < 0.001). After adjustment for covariates, a low PLGF concentration still significantly increased the risk of developing discordant fetal growth (monochorionic twin pregnancy: adjusted OR: 7.039, 95% CI: 2.798–17.710, *P* < 0.001; dichorionic twin pregnancy: adjusted OR: 4.279, 95% CI: 2.572–7.120, *P* < 0.001). Therefore, a low PLGF level can be considered an independent predictor of the risk of discordant fetal growth, regardless of maternal age, gestational weight gain, or gestational age at delivery.

## Discussion

Accurate prenatal prediction and recognition of discordant fetal growth are critical for deciding suitable management strategies. However, although intensive fetal surveillance using prenatal ultrasonography has been associated with the prediction and recognition of discordant fetal growth, the extent to which labor-intensive surveillance is effective in clinical practice is still controversial [8, 21]. Moreover, studies on fetal biometric parameters for predicting discordant growth in twins have reported inconsistent results regarding accuracy [22–25]. Therefore, novel biomarkers for discordant fetal growth are urgently required despite the considerable challenges.

In this study, the twin-pregnant women were divided into two groups according to chorionicity based on two considerations. First, the pathophysiology of discordant growth in monochorionic twins differs from that in dichorionic twins. The unequal distribution of placental masses supplying the two fetuses, different types of placental anastomotic vessels, and different insertion sites of the umbilical cord induce discordant growth in monochorionic twins. In contrast, different genetic potentials, locations of placentation, and placental dysfunction are major contributors to dichorionic growth [26–28]. In this study, different characteristics were observed between monochorionic and dichorionic twins. In monochorionic twins, newborns with discordant growth had a lower birth weight in smaller and larger fetuses, lower 1- and 5-min Apgar scores, and higher incidence of NRDS. However, in dichorionic twins, no significant differences were observed in the birth weight of the larger fetus or 1-min Apgar score. Consistent with most of previous studies’ findings, our findings suggested that monochorionic twins have a higher risk of adverse neonatal outcomes [29, 30]. Second, maternal second-trimester PLGF levels may vary depending on chorionicity, given that PLGF is produced by villous syncytiotrophoblasts in the placenta. Based on the fact that placental volumes differ between monochorionic and dichorionic twin pregnancies, maternal second-trimester PLGF levels vary. This is supported by the results of several previous studies [31, 32].

Based on the different chorionicity, we compared maternal second-trimester PLGF levels between women who subsequently developed discordant fetal growth and those who did not. Surprisingly, a 3–4 fold difference in the median PLGF concentration was observed between the two groups in both monochorionic and dichorionic pregnancies. Lower PLGF levels suggest that patients who subsequently developed discordant fetal growth might have already experienced underlying placental dysfunction in the second trimester, regardless of the clinical manifestations. For the first time in literature, our research demonstrated significant correlations between maternal second-trimester PLGF levels and birth weight differences in twin pairs. These findings have increased the potential of using maternal second-trimester PLGF levels to predict discordant fetal growth.

To further confirm the predictive value of maternal second-trimester PLGF levels, an ROC curve analysis was performed. The AUC was calculated as 0.751 (95% CI: 0.649–0.852) and 0.716 (95% CI: 0.655–0.777) for monochorionic and dichorionic twin pregnancies, respectively. In general, the predictive efficiency was considered moderate. In several previous studies, the predictive value of fetal biometric parameters obtained using ultrasonography was consistent with that in our results. Loppke FB et al. (*n* = 762) revealed that CRL discordance can be used to predict discordant fetal growth with an AUC of 0.70 (95% CI: 0.63–0.76) in monochorionic twin pregnancies [25]. In another study by Ting Yuan et al., which included 417 dichorionic twin pregnancies, the AUCs of BPD, HC, AC, FL, and EFW in the late third trimester were reported to be 0.634, 0.617, 0.683, 0.751, and 0.759, respectively [10]. Considering that the prediction of discordant fetal growth is currently limited to ultrasound parameters and that a few studies have focused on maternal serum biomarkers, the detection of the maternal second-trimester PLGF level is a promising strategy for the prediction of discordant fetal growth, although its predictive performance is moderate [20, 33]. With the development of detection technologies, the maternal serum PLGF level can be detected quickly and automatically in clinical laboratories, offering important advantages over labor-intensive ultrasonography.

Meanwhile, the OR of PLGF levels was calculated in the crude regression models and then adjusted for potential confounding factors, including a maternal age of > 30 years, gestational weight gain, and gestational age at delivery. Consistent with most of the previous reports’ findings, the difference in maternal age between the discordant growth and normal groups was not significant in both monochorionic and dichorionic pregnancies when categorized by 35 years (monochorionic twin pregnancy: *P* = 0.789; dichorionic twin pregnancy: *P* = 0.998) [19, 22, 34]. However, the difference became significant when categorized by 30 years (monochorionic twin pregnancy: *P* = 0.044; dichorionic twin pregnancy: *P* = 0.027). Specifically, twin-pregnant women with a maternal age of > 30 years are at a higher risk of developing discordant fetal growth. These findings suggest that maternal age is a potential factor responsible for discordant fetal growth, although this threshold requires further research. We calculated the adjusted ORs for maternal serum PLGF levels based on these aforementioned potential confounding factors. The adjusted ORs indicated that when the second-trimester PLGF concentration is of < 187.5 pg/mL (monochorionic twin pregnancy) or of < 252.5 pg/mL (dichorionic twin pregnancy), the risk of developing discordant fetal growth is increased by 7.039 or 4.279 times, respectively. These results further confirm that a low PLGF level can be used as a stable predictor of the risk of developing discordant fetal growth.

The strength of this study is that it provides maternal serum PLGF data for twin-pregnant women based on chorionicity, identifying those at a high risk of developing discordant fetal growth using a novel method, thereby allowing them to benefit from the intensive surveillance. The detection of the PLGF level provides a complementary screening strategy for the prediction of discordant fetal growth and offers a unique perspective for subsequent research in this field. In addition, we can reassure twin-pregnant women who have a small discordance shown via ultrasound parameters regarding the risk of discordant fetal growth, since in our study, more than 90% of twin-pregnant women did not develop discordant fetal growth when second-trimester PLGF concentrations were above the cutoff values.

This study had several limitations. First, it only included maternal second-trimester PLGF levels and did not involve early- and late-trimester data; thus, it was impossible to conduct a longitudinal study throughout the pregnancies. Second, we used a single maternal serum biomarker. However, the inclusion of additional biomarkers, such as soluble fms-like tyrosine kinase-1 (sFlt-1), would allow for a more comprehensive evaluation. Third, the development of predictive models that combine maternal serum biomarkers and fetal biometric parameters may improve the predictive accuracy of discordant fetal growth, which we may focus on in our future studies. Finally, the retrospective nature of this study inherently brings some inevitable limitations, such as limited access to potential confounders and missing data. In this study, the information for each participant was retrieved from the medical information database, in which the ultrasound parameters and Doppler fetal flussimetry were incomplete. Therefore, we were unable to include them in the statistical analysis, although it is indeed a very interesting study.

## Conclusions

This study showed that maternal second-trimester PLGF levels are significantly correlated with birth weight differences in both monochorionic and dichorionic twin pregnancies. A low maternal second-trimester PLGF level can be considered a remarkable risk factor and potential predictor of discordant fetal growth. The detection of the PLGF level can provide a complementary screening strategy to predict discordant fetal growth, although more prospective multi-indicator studies are required.

## Data Availability

The data supporting this study’s findings are available on request from the corresponding author. The data are not publicly available due to privacy or ethical restrictions.

## Abbreviations

PLGF: Placental growth factor
ROC: Receiver operating characteristic
OR: Odds ratio
CI: Confidence interval
CRL: Crown-lump length
BPD: Biparietal diameter
HC: Head circumference
AC: Abdominal circumference
FL: Femur length
EFW: Estimated fetal weight
BMI: Body mass index
NRDS: Neonatal respiratory distress syndrome
